# A new method for estimating growth and fertility rates using age-at-death ratios in small skeletal samples: The effect of mortality and stochastic variation

**DOI:** 10.1371/journal.pone.0286580

**Published:** 2023-06-02

**Authors:** Patrik Galeta, Anna Pankowská

**Affiliations:** Department of Anthropology, University of West Bohemia, Pilsen, Czech Republic; University of Michigan, UNITED STATES

## Abstract

The common procedure for reconstructing growth and fertility rates from skeletal samples involves regressing a growth or fertility rate on the age-at-death ratio, an indicator that captures the proportion of children and juveniles in a skeletal sample. Current methods derive formulae for predicting growth and fertility rates in skeletal samples from modern reference populations with many deaths, although recent levels of mortality are not good proxies for prehistoric populations, and stochastic error may considerably affect the age distributions of deaths in small skeletal samples. This study addresses these issues and proposes a novel algorithm allowing a customized prediction formula to be produced for each target skeletal sample, which increases the accuracy of growth and fertility rate estimation. Every prediction equation is derived from a unique reference set of simulated skeletal samples that match the target skeletal sample in size and assumed mortality level of the population that the target skeletal sample represents. The mortality regimes of reference populations are based on model life tables in which life expectancy can be flexibly set between 18 and 80 years. Regression models provide a reliable prediction; the models explain 83–95% of total variance. Due to stochastic variation, the prediction error is large when the estimate is based on a small number of skeletons but decreases substantially with increasing sample size. The applicability of our approach is demonstrated by a comparison with baseline estimates, defined here as predictions based on the widely used Bocquet-Appel (2002, doi: 10.1086/342429) equation.

## Introduction

The fluctuation of fertility and growth rates is viewed both as a cause and consequence of fundamental transformations within human societies [1–6]. Population pressure has been seen as a contributing factor to general cultural evolution, including major transitions such as the origin of agriculture, agricultural intensification, early political evolution, the origin of warfare, and industrialization [7–10]. An example of such an interaction is the model of the Neolithic demographic transition [but see, 11]. According to this model, fertility increased substantially during the transition from foraging to farming, which resulted in the rapid growth of early Neolithic populations [8, 12, 13]. Signals of this transition have been detected in prehistoric Europe [12, 14, 15], North America [16, 17], Mesoamerica [18], the Levant [19], and East and Southeast Asia [20].

On a methodological level, a common procedure for estimating growth and fertility rates in skeletal samples involves the regression of the demographic variables on the ratios of two broad age-at-death categories. Bocquet-Appel [12], for example, proposed the D5–19/D5+ ratio (called P index), which compares the number of non-adults who died between the ages of 5 and 19 years with the total number of deaths aged 5+. The P index is a good predictor of growth and fertility levels, since children are relatively more numerous in growing populations compared to stationary and decreasing populations. However, it has recently been argued [21] that the P index is more responsive to variation in age-independent mortality rather than as an indicator of fertility. Several different age-at-death ratios capturing the proportion of non-adult skeletons in skeletal samples have been published to date. Bocquet-Appel and Masset [22] proposed the D5–14/D20+ ratio (the juvenility index), Buikstra et al. [23] presented the D30+/D5+ ratio, McFadden, Oxenham [24, 25] the D0–14/D ratio, Belwood and Oxenham [20] the D20+/D5+ ratio, Larsen et al. [26] the D3–19/D3+ ratio, and Robbins [27] the D0/D2–19 ratio.

The advantage of the age-at-death ratios in estimating growth and fertility levels is that they overcome two important limitations of paleodemographic research: the error in estimating age-at-death from skeleton and the underrepresentation of children in archaeological record [1, 28]. The age-at-death estimation has especially low precision in adults, and the reported age-at-death interval often encompasses decades [29]. The ratios minimize this error because individuals are combined into two broad groups (e.g., D20+, D5+) [28, 30]. Some of the ratios ignore the deaths of newborns and the youngest children, which helps to address the issue of their poor representation in the archaeological record due to differential skeletal decomposition [31], recovery [32], and cultural practices [24, 33, 34].

Despite the general benefits of the use of age-at-death ratios in estimating growth and fertility rates, there are challenges with this approach. The existing ratio methods [e.g., 12, 24] predict growth and fertility rates using regression equations that were derived from modern populations and then applied to prehistoric populations, whose mortality levels may not match those of the modern reference. Bocquet-Appel [12] built his regression formula on a reference set of pre-industrial populations with life expectancy at birth of 18–38 years and applied them to predict a growth rate in Neolithic populations whose life expectancies at birth were considerably lower and likely did not exceed 25 years [8]. McFadden and Oxenham [24, 25] derived formulas for predicting growth and total fertility rate in bioarchaeological sites based on a reference set of raw age-at-death data from modern countries, in which life expectancy at birth is between 42 and 74 years. These applications of modern reference to prehistoric populations benefit from the principle of uniformitarianism [35, 36] (which postulates continuity between demographic processes and their causal mechanisms) but go beyond the principle by assuming that the regression relationship between age structure of deaths and growth rate is the same at different mortality levels.

Another methodological concern is that the ratio methods do not reflect the effect of random error on the age distribution of deaths. The prediction equations have been derived from populations with a large number of deaths in which an effect of stochastic variation on age-at-death distribution and ratios is small or negligible. Stochastic variation may be a substantial issue in real paleodemographic settings where skeletal samples are typically small, with only tens or a few hundred skeletons. For example, 72% out of 68 skeletal samples used in the Bocquet-Appel’s [12] Neolithic dataset had less than 100 skeletons, and several sites consist of fewer than 20 individuals.

The age structure of deaths may differ substantially between two small samples even if they represent the same population. The proportion of skeletons aged 5–19 years, for example, can vary by chance from 4 to 19% in samples with 100 skeletons, although all these samples are derived from a population where individuals between 5 and 19 years account for 10% of deaths (assuming the mortality pattern described by the Coale and Demeny’s [37] West model table with life expectancy at birth of 25 years and zero annual growth rate; the procedure for generating skeletal samples is described in the Methods section). The most susceptible to stochastic variation are narrow age-at-death categories (such as D5–14 of the juvenility index [22]) as any accidental death can have a profound effect on the age-at-death ratio, which impairs the prediction of demographic indicators [38].

In this study, we propose a new methodology for estimating growth, birth, and fertility rates using age-at-death ratios. The procedure accounts for stochastic variation in small-sized skeletal samples and supports flexible settings of mortality level of reference populations. Both additions increase the accuracy of the demographic prediction. We offer a general simulation algorithm that allows a unique prediction equation to be created for each real skeletal sample. Each equation is derived from a unique set of reference skeletal samples that have the same size as the skeletal sample under study and are derived from populations with mortality levels appropriate for the time period being studied. We also present three new age-at-death ratios that minimize the problem of ageing errors from skeleton, and the underrepresentation of infants in the archaeological record. Finally, we compare growth and crude birth rate estimations using the proposed method with baseline values, defined here as estimates based on the widely used Bocquet-Appel’s [12] P index.

## Materials and methods

### Age-at-death ratios used in demographic estimation

We propose three new age-at-death ratios: D5+/D20+, D3+/D20+, and D1+/D20+. The ratios compare the number of skeletons older than 5, 3, and 1 years, respectively, to the number of adult skeletons aged 20 years and over. The ratios reflect an elevated proportion of children and juveniles, and are therefore higher in growing, highly fertile populations than in stationary or declining ones. Note that all three ratios omit infants and youngest children, who are usually underrepresented in the burial record, and use the age-at-death demarcation points that can be reliably recognized on the skeleton.

If an individual’s age-at-death cross cuts several age-at-death categories, the individual is proportionally distributed among two or more categories [17]. For example, an individual whose age-at-death is estimated to lie between 4–8 years (i.e., 4.0–8.9 years) is divided into two parts: 0.2 of the individual is assigned to the D0–4 group and the remaining 0.8 of the individual assigned to the D5+ group. The age-at-death adjustment accounts for natural variability between biological and chronological age [1] and is superior to using the point estimate (the midpoint of 6 years) and assigning the individual to the D5+ group as a single unit.

### Algorithm for predicting growth, birth, and fertility rates

**Fig 1:**
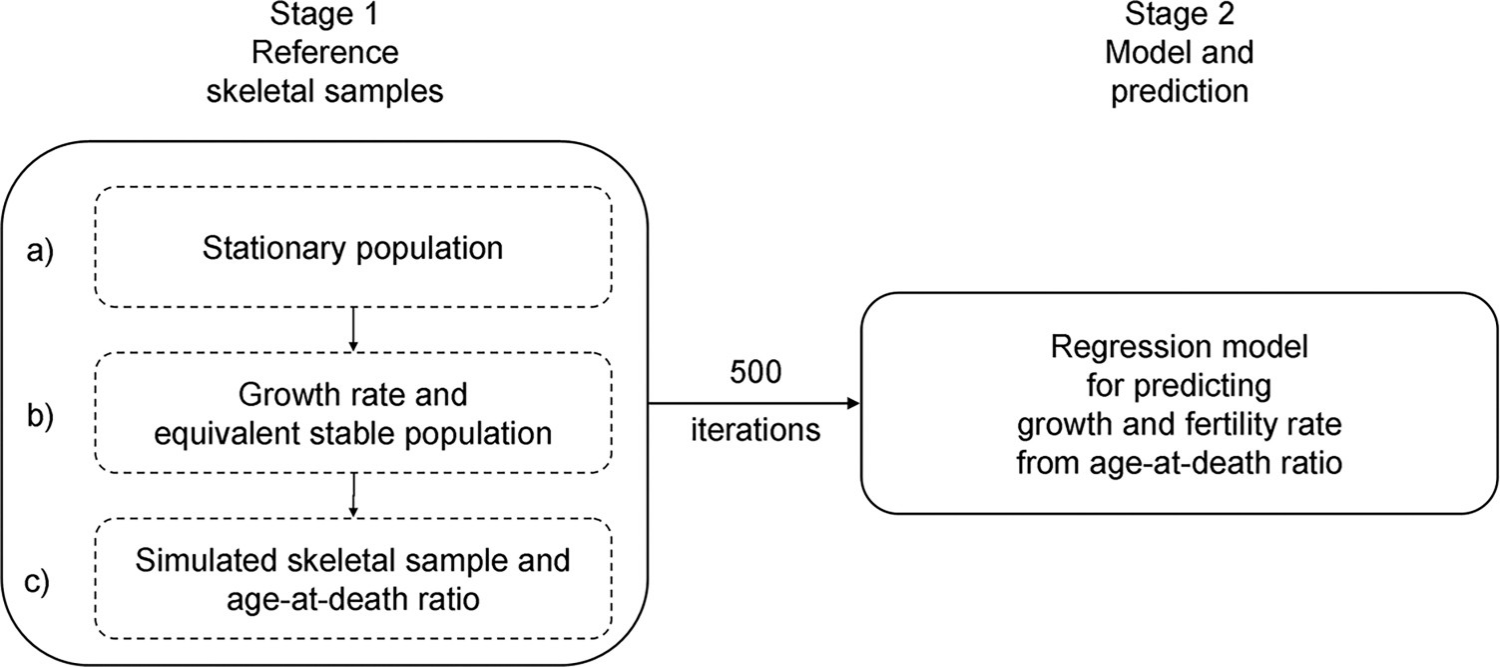
Outline of the proposed algorithm for predicting the demographic characteristics of a population represented by a skeletal sample using age-at-death ratios.

The algorithm for estimating demographic indicators from the age-at-death ratio involves two stages (Fig 1). In the first stage, a reference set of skeletal samples drawn from populations with known demographic characteristics is produced. In the second stage, the reference set is used to build regression models for predicting demographic indicators of the population represented by the skeletal sample under study.

Unlike the previous ratio methods, the estimation of demographic characteristics for each real skeletal sample is based on a unique set of reference skeletal samples that match the size and the mortality level of the real skeletal sample under study. Such a strategy incorporates the stochastic variation of the age distribution of deaths as it allows learning about how the age-at-death ratios behave in samples of the same size as the real skeletal sample.

#### Stage 1: Reference set of skeletal samples

Given that there are no skeletal samples with known demographic characteristics dating back to before ca.18^th^ century AD, reference skeletal samples are created by simulation. Each reference skeletal sample (i.e. raw deaths) is generated following a three-step procedure (Fig 1): (a) computing a life table for the reference stationary population, (b) setting the growth rate and computing the stable equivalent population, and (c) sampling deaths from a stable equivalent population to obtain a reference skeletal sample of a given size and computing age-at-death ratios. The total number of reference skeletal samples created by iterating these three steps of Stage 1 should be between 500 and 1000.

*(a) Life table for reference stationary population*. The mortality patterns of reference stationary populations are based on Coale and Demeny’s West model life tables [37]. The Coale and Demeny system is thought to be appropriate for modeling historical and prehistoric populations [39, 40] and has been used as a general pattern where there is no reliable guide to the age pattern of mortality that prevails [15, 37, 41–43]. The Coale and Demeny system is organized into 25 levels according to female life expectancy at birth. The levels are evenly distributed between Level 1 (life expectancy of 20 years) and Level 25 (life expectancy of 80 years), with increments of 2.5 years [37].

A life table for reference stationary populations is computed from the proportion of those surviving at exact age x (l_x_ column of Levels 1–25) using a standard demographic algorithm [44]. Since Coale and Demeny tables are abridged, Siler’s five-parameter competing-risk mortality model is applied to obtain the survivorship column for the complete life tables. The coefficients of the Siler model for odd-numbered tables are adopted from Gage and Dyke [45], and the coefficients of the remaining tables are approximated using polynomial regression. Such an approximation allows the construction of 63 complete Coale and Demeny life tables with the life expectancy between 18 and 80 years with a step of one year. In each iteration, one life table is randomly chosen and processed further.

*(b) Growth rate and stable equivalent population*. In every iteration, a value of the intrinsic rate of natural increase (growth rate) is set at random from the interval between −3% and 3% per annum (assuming uniform distribution) and applied to the life table of the reference stationary population (step a). The number of deaths aged x, crude birth rate, and net reproduction rate are calculated according to Lotka’s equation for the stable population [44]. The total fertility rate is then approximated with the net reproduction rate divided by the proportion of births who are girls (100 girls per 205 births) multiplied by the probability of a woman surviving to the mean age at childbearing (27.5 years) [46] derived from the stationary life table. The proportion of births who are girls and the mean age at childbearing are assumed to be relatively stable among human populations with natural reproduction [46].

*(c) Simulated reference skeletal sample and the age-at-death ratios*. Each simulated skeletal sample (i.e., a sample of deaths) is derived by consecutive uniform random sampling between 0 and 1, and binning them according to the cumulative age distribution of deaths in the stable equivalent population (step b) scaled to sum up to one [47]. The scaled cumulative age distribution of deaths measures the probability that a randomly selected individual dies at the age of x or younger. If the cumulative age distribution of deaths at ages 0, 1, and 2 equals, for example, 0.46, 0.53, and 0.55, respectively then an individual selected at random has a 46% chance of dying as an infant, a 53% chance of dying at the age of one year or younger, and 55% chance of dying at the age of two years or younger. Three randomly selected numbers (0.30, 0.45, and 0.54, for example) mean that the first two simulated individuals died as infants and the third individual died at age 2. A simulated skeletal sample is produced with the same number of adult skeletons (D20+) as the real skeletal sample under study. Finally, the corresponding number of skeletons in the D1+, D3+, D5+, and D20+ age-at-death groups are computed and used to calculate the three proposed age-at-death ratios.

The three-step procedure is repeated 500–1000 times (see above) to obtain the reference set of simulated skeletal samples. Note that although the number of adults is fixed, the total number of skeletons varies across simulated samples because they are drawn from stable populations with different levels of population growth. In samples with 50 adults for example, the total number of individuals can range from 55 individuals in populations declining by 3% per year to ca. 400 individuals in populations increasing by 3% per year.

#### Stage 2: Regression models for predicting growth, birth, and fertility rates

The reference set of simulated skeletal samples is used to build univariate regression models for predicting the growth rate (intrinsic rate of natural increase in percent per annum), crude birth rate (CBR, the annual number of live births per 1,000 population), and total fertility rate (TFR, average number of children born per woman during her childbearing period) of the population buried at the cemetery under study. In the regression models, one of the demographic indicators (growth rate, CBR, or TFR) is used as the dependent variable, whereas one of the age-at-death ratios (D5+/D20+, D3+/D20+, or D1+/D20+) is considered a predictor. A non-linear generalized additive models (GAM) regression with cubic splines was found to best fit the data in preliminary simulations because it allows for the flexible specification of the dependence of the response on the predictor variable [48]. To reduce skewness in the distribution of the data and to improve the predictive ability of the model, logarithmic transformation is applied to the independent variable in the models predicting growth rate and crude birth rate, and to the dependent variable in the model predicting total fertility rate, respectively.

#### Flexible settings of the algorithm inputs

A simulation algorithm was programmed in R [49] (S1 Text). It supports flexible settings of three input parameters, which allows generating the reference set of skeletal samples that match the size of a skeletal sample under study (D20+) and the demographic regime of a reference population that a target skeletal sample represents. These parameters include the mortality level of reference populations (life expectancy at birth can be set between 18 and 80 years) and their annual growth rate (can be set between −3 and 3%). The third flexible input is the total number of reference skeletal samples to be drawn (should be higher than 500).

The main outputs of the algorithm are the predicted growth, crude birth, and total fertility rates with their 95% prediction intervals. The algorithm further outputs distributions of the age-at-death ratios in the reference sets of simulated skeletal samples, which may serve as a measure of skeletal sample bias. If the age-at-death ratio observed in the target skeletal sample lies outside its distribution in the reference skeletal samples, the age-at-death composition of a target sample may be biased, and the prediction of demographic rates should be avoided (see [50] for another approach to detecting samples with biased distributions of deaths).

### Comparison with predictions based on Bocquet-Appel’s P index

To show the applicability of the proposed algorithm, the D5+/D20+ ratio was used to predict the growth and crude birth rates in the skeletal samples of Bocquet-Appel’s [12] dataset and then compared with the estimations based on P index and calculated from the original Bocquet-Appel’s equation [12, p. 643]. The Bocquet-Appel’s estimates are used as a baseline for a comparison because his method is the most widely used for predicting growth rates from skeletal samples. However, his estimates do not represent “true” population growth rates.

Growth rate prediction based on the D5+/D20+ ratio was calculated using our algorithm, which was set to generate reference sets of 500 simulated skeletal samples that were drawn from reference stationary populations with life expectancies between 18 and 25 years and subjected to an annual growth between −3 and 3% per annum. To allow the comparison, we followed Bocqet-Appel [12] and kept mortality (life expectancy at birth) within the same range for all samples although the assumption of unchanged mortality during the entire Neolithic Demographic Transition is not realistic [51]. The more appropriate prediction would take into account a mortality level of each individual population, which, unfortunately, cannot be done from Bocquet-Appel’s summary data [12, pp. 640–641]. Our other ratios (D3+/D20+ and D1+/D20+) were not used for comparison, because Bocquet-Appel did not publish data that would allow their computation.

The comparison of the growth and crude birth rate estimation based on the D5+/D20+ ratio and the P index was made using Bland and Altman’s [52] approach to measure agreement between two methods by studying the mean difference (MD) and constructing limits of agreement. MD estimates the magnitude of the systematic difference (bias) between two methods, and it is computed as the mean of the difference between estimates based on the D5+/D20+ ratio and P index. The limits of agreement represent the range around the MD in which 95% of the differences between two estimates are expected to be found, and are computed as MD±1.96 standard deviation of the difference.

Bocquet-Appel’s [12] dataset consists of 68 Mesolithic and Neolithic skeletal samples and was originally used to reveal a signal of Neolithic demographic transition. The samples are geographically distributed across Europe; two samples are located in the adjacent region of North Africa [12, p. 638]. The numbers of skeletons in the 0–4, 5–19, and 20+ age-at-death categories and the values of the P index were adopted from Bocquet-Appel’s study [12, pp. 640–641]. Five Bocquet-Appel’s samples had to be omitted from the comparison because estimation results showed that their age-at-death ratios may be biased (ratio values were outside their distribution in the simulated reference skeletal samples). The comparison between estimations based on the D5+/D20+ ratio and the P index was finally based on 63 Bocquet-Appel’s samples with the total number of skeletons between 11 and 216 individuals and a median of 64 individuals.

## Results

### Regression models for predicting growth and fertility rates

**Fig 2:**
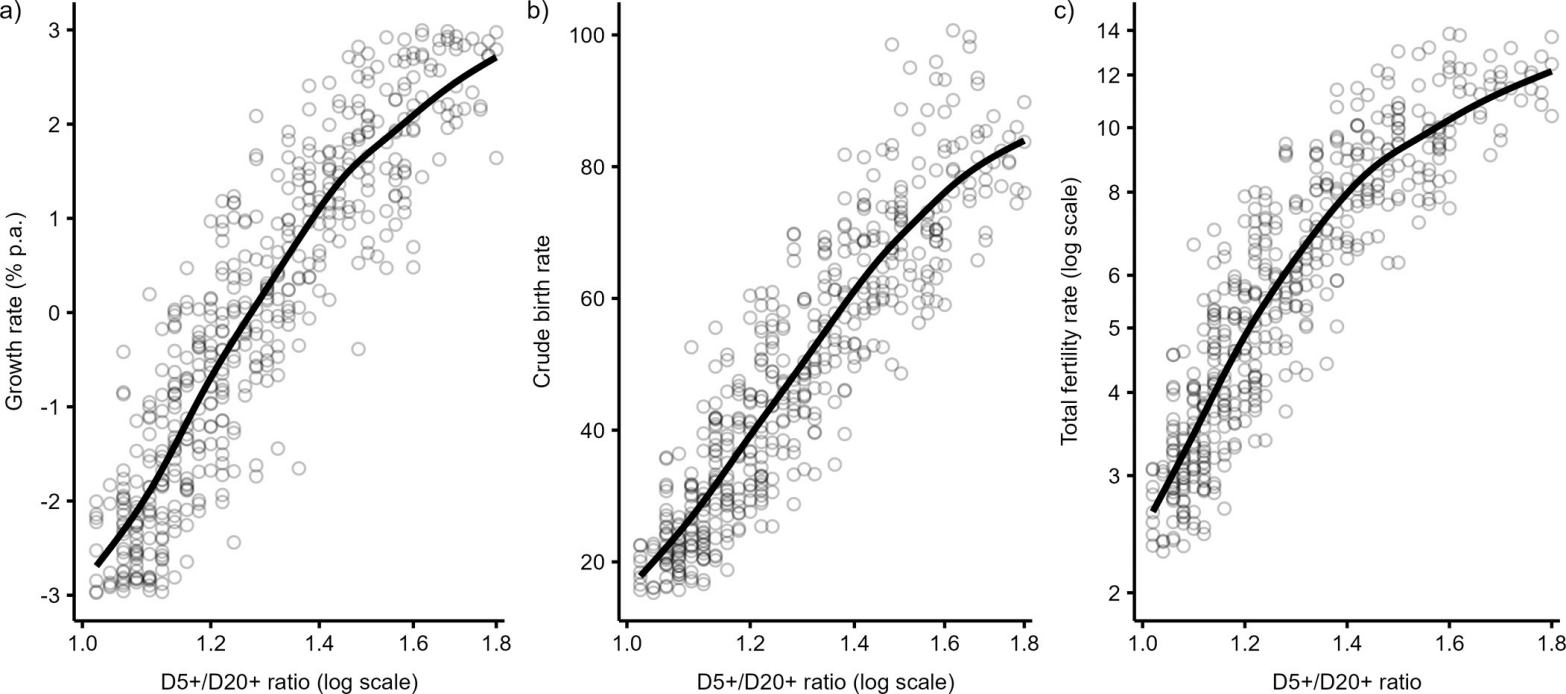
Regression fits between the D5+/D20+ ratio and (a) the growth rate, (b) the crude birth rate, and (c) the total fertility rate. Generalized additive regression models (solid lines) are based on 500 simulated reference skeletal samples (circles) with 50 adult skeletons (D20+ = 50) randomly drawn from populations with a life expectancy between 18 and 25 years and annual growth rate between −3 and 3%.

The regression models between the demographic indicator and age-at-death ratio are statistically significant (P<0.001). Fig 2 shows the scatterplots and regression fits between (a) the growth rate, (b) the crude birth rate, and (c) the total fertility rate and the D5+/D20+ ratio as predictor to document these relationships. According to adjusted *R*-squared, the D5+/D20+ ratio successfully explains 83–84% of the corresponding demographic indicator. A high proportion of explained variance allows the reliable prediction of the demographic parameters of population from which the skeletal sample was drawn.

Table 1 summarizes the basic characteristics of regression models based on various combinations of age-at-death ratio and the number of adults in a skeletal sample. It documents that the amount of explained variability increases and prediction error decreases with the size of the skeletal sample. The explanation ability of the models based on the D5+/D20+ ratio is 66–67% in small samples with only 25 adult skeletons but increases to 89–90% in larger samples with 100 adult deaths. Growth rates can be predicted within the error of ±1.9%, ±1.4%, and ±1.1% in samples with 25, 50, and 100 adult skeletons, respectively. Further, regression models with the D3+/D20+ and the D1+/D20+ ratios explain more variance and have lower prediction errors than models based on the D5+/D20+ ratio. The best results are achieved by a regression fit based on the D1+/D20+ ratio calculated in large skeletal samples with 100 adults, which explains, for example, 94% of the variance in growth rate and allows its prediction within ±0.9% per annum.

**Table 1:** Characteristics of univariate regression fit between three age-at-death ratios and three demographic variables based on skeletal samples with three different numbers of adults (D20+).

			D20+ = 25 ind.		D20+ = 50 ind.		D20+ = 100 ind.	
Dependent (demographic) variable	Predictor (ratio)	Ratio value	Adj. *R*^2^	95% prediction error	Adj. *R*^2^	95% prediction error	Adj. *R*^2^	95% prediction error
Growth rate (% per annum)	D5+/D20+		0.66	±1.9	0.83	±1.4	0.89	±1.1
	D3+/D20+		0.70	±1.8	0.86	±1.3	0.91	±1.0
	D1+/D20+		0.82	±1.4	0.90	±1.1	0.94	±0.9
Crude birth rate (CBR, live births per 1,000 pop.)	D5+/D20+		0.67	±24	0.83	±17	0.90	±13
	D3+/D20+		0.71	±22	0.85	±16	0.92	±12
	D1+/D20+		0.83	±17	0.91	±13	0.95	±9
Total fertility rate (TFR, children born per woman)	D5+/D20+	1.14	0.66	±2.4	0.84	±1.6	0.90	±1.1
		1.26		±3.2		±2.3		±1.8
		1.44		±4.9		±3.4		±2.7
	D3+/D20+	1.18	0.71	±2.2	0.86	±1.4	0.92	±1.1
		1.33		±3.0		±2.1		±1.6
		1.56		±4.5		±3.1		±2.4
	D1+/D20+	1.34	0.83	±1.6	0.92	±1.1	0.95	±0.8
		1.64		±2.3		±1.6		±1.2
		2.22		±3.6		±2.4		±1.9

Note: Adj. *R*^2^: adjusted *R*-squared; D20+, number of skeletons older than 20 years; ind.: individuals.

Ratio values represent lower quartile, median, and upper quartile of their respective distribution in simulated skeletal samples with 50 adult skeletons that are drawn from populations with life expectancies at birth between 18 and 25 years and with annual growth rate between −3 and 3%.

Adjusted *R*-squared represents the proportion of variability in the demographic indicator that is explained by the model.

95% prediction error (half of the prediction interval) represents the uncertainty in the fit and means that one has a 95% chance that the predicted demographic indicator value is actually contained within its bounds.

**Fig 3:**
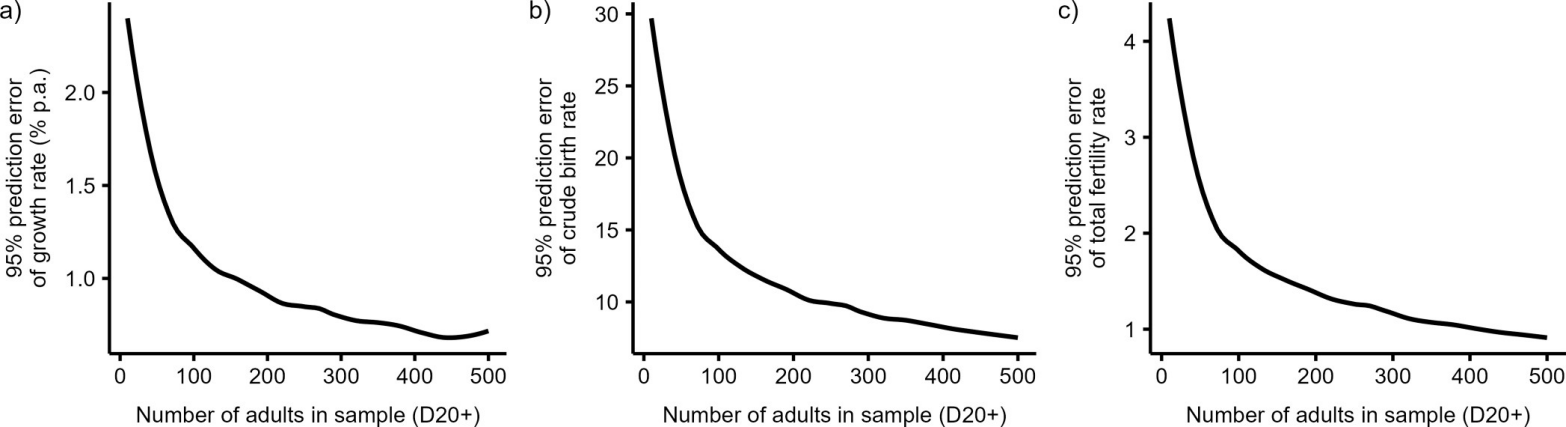
Relationship between the size of skeletal sample (measured as number of adults, D20+) and 95% prediction error of (a) the growth rate, (b) the crude birth rate, and (c) the total fertility rate. Prediction errors (half of the 95% prediction interval) are calculated from regression models using the D5+/D20+ ratio as predictor and based on 500 simulated skeletal samples randomly drawn from populations with a life expectancy at birth between 18 and 25 years and annual growth rate between −3 and 3%. Predictions are made for the D5+/D20+ ratio of 1.26.

Fig 3 further shows how the 95% prediction error decreases with the increasing size of skeletal samples. For example, the prediction of growth rate (Fig 3A) is very imprecise (±2.5%) for extremely small samples with only 10 adult skeletons. In larger samples, the prediction improves rapidly. Based on samples with 100 and 500 adults, growth rates can be predicted within a margin of ±1.13% and ±0.70%, respectively. The margin of prediction error of the total fertility rate is again high in small samples (±4 children per woman), then decreases substantially in samples with ca. 80 adult skeletons (±2 children per woman) and attains ±0.5 children per woman in skeletal samples with 500 adults.

We do not present the regression equation of any particular model here, since estimation for each real skeletal sample is based on a unique set of reference skeletal samples, which is used to build a unique regression model.

### Comparison with predictions based on Bocquet-Appel’s P index

**Fig 4:**
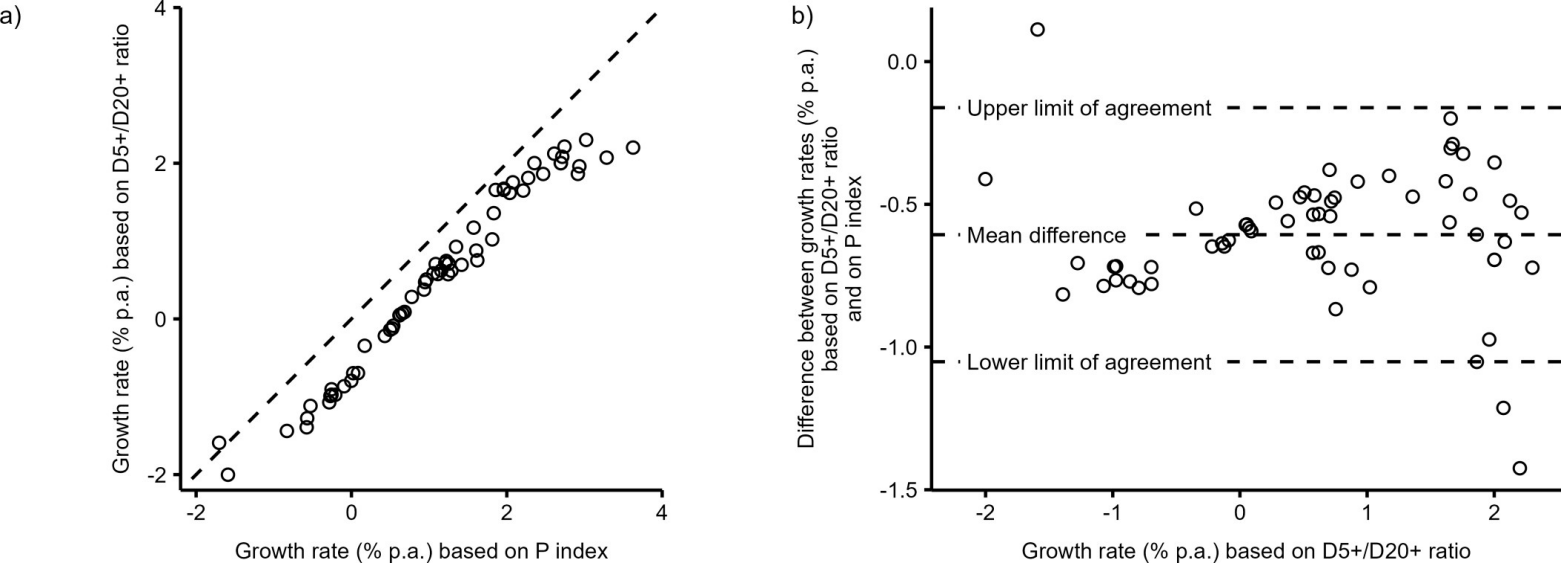
Comparison of annual growth rate (%) predicted using D5+/D20+ ratio and Bocquet-Appel’s [12] P index in 63 Mesolithic and Neolithic skeletal samples (circles) [12, pp. 640–641]. (a) Scatterplot comparing two growth rate estimates; the identity line is dashed; (b) Bland and Altmann plot showing the mean difference and limits of agreement between the two methods of estimation.

The estimations of the growth, birth, and fertility rates for skeletal samples of Bocquet-Appel’s [12] dataset based on both the D5+/D20+ age-at-death ratio and the P index are shown in S1 Table. The correlation between growth rate estimations based on the D5+/D20+ ratio and on Bocquet-Appel’s P index (Fig 4A) is excellent (Pearson *r* = 0.98, P<0.001, *n* = 63 skeletal samples). The mean difference (MD = −0.61% per annum) demonstrates that growth rates based on the D5+/D20+ ratio are systematically lower than those based on the P index. The limits of agreement show that 95% of differences lie in the range of −1.05 to −0.16% per annum. An examination of Fig 4B suggests, however, that differences between two methods are not randomly scattered across the growth rate range. With few exceptions at both ends, the difference between the growth rate estimates based on the D5+/D20+ ratio and those based on the P index decreases with increasing growth rate.

**Fig 5:**
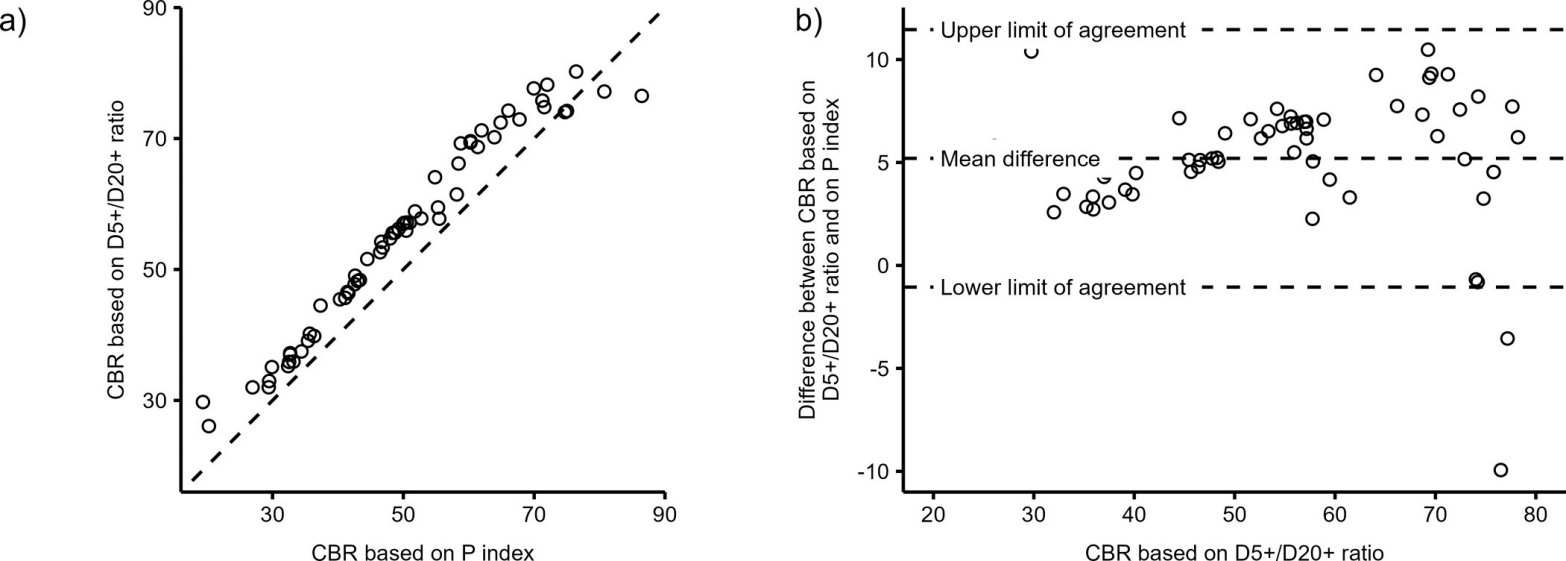
Comparison of crude birth rate (CBR, live births per 1,000 population) predicted using the D5+/D20+ ratio and Bocquet-Appel’s [12] P index in 63 Mesolithic and Neolithic skeletal samples (circles) [12, pp. 640–641]. (a) Scatterplot comparing two CBR estimates; the identity line is dashed; (b) Bland and Altmann plot showing mean difference and limits of agreement between the two methods of estimation.

A similar comparison of the two methods for the crude birth rate is presented in Fig 5. Predictions based on the D5+/D20+ ratio are systematically higher than those based on P index across the full range of CBR values (MD = 5.2 live births). Differences between the two methods become greater with increasing CBR values; they are around 3 units for CBR around 30–40 live births and reached values around 8 units when one estimates CBR around 70 live births. According to the limits of agreement, 95% of differences are within −1.0 and 11.5 units.

## Discussion

### New age-at-death ratios

The proposed age-at-death ratios (D5+/D20+, D3+/D20+, and D1+/D20+) have, like the Bocquet-Appel’s [12] P index, several benefits in paleodemographic analysis. The ratios minimize the issue of age-estimation error, as all non-adults and all adults are combined to form two large groups. Individuals can be confidently placed into these two age-at-death groups since only two demarcation points need to be distinguished on the skeleton. The age point of 5 year (or 3 and 1 years, respectively), is easily recognized on the skeleton based on the chronology of tooth mineralization and eruption, and the point of 20 years is manifested by attaining skeletal maturity [53]. The ratios eliminate newborns and infants, the most underrepresented group in the cemetery record, which limits paleodemographic analysis based on the classic life table approach [54]. The D3+/D20+ and D5+/D20+ ratios further omit the youngest children up to 2 and 4 years of age, respectively, and should be more suitable for samples in which differential preservation, recovery, and cultural customs were not limited only to infants.

The definition of the new age-at-death ratios follows the pioneering works by Bocquet-Appel and Masset [12, 22]. In fact, our D5+/D20+ ratio uses the same two age-at-death demarcation points (5 and 20 years) as Bocquet-Appel’s P index (D5–19/D5+ ratio), and both indicators can be transformed into each other. The D3+/D20+ and D1+/D20+ ratios are then an extension of the basic D5+/D20+ ratio.

Although the youngest individuals are excluded from the computation our age-at-death ratios, they correlate closely with the growth, crude birth, and total fertility rates (adjusted *R*-squared>0.83 and 0.89 for samples with more than 50 and 100 adult skeletons, respectively, Table 1). Our regression models explain more variance of demographic indicators than models using the D0–14/D ratio, although this ratio incorporates all skeletons, including infants (*R*-squared 0.72 and 0.74 for models predicting total fertility rate [24] and growth rate [25], respectively). However, the closer relationship of our models may also be due the regression method, because polynomial regression used here fits the data closer to the regression line than the linear regression used in [24, 25]. By contrast, the margin of error of model predicting the total fertility rate based on the D0–14/D ratio (ca. ±2 children [24, p. 477]) is similar or smaller than the prediction error of our models based on the D5+/D20+ ratio (±1.6–3.4 children, assuming samples with 50 adults, Table 1). The D0–14/D ratio may therefore be a preferred proxy for fertility rates in sites with excellent preservation of skeletons and good infant representation [24, 28, 55].

### Effect of mortality on prediction of growth rate

A new feature of the proposed approach is the flexibility of setting the mortality levels of reference populations (Stage 1a of the algorithm, Fig 1). The choice of reference mortality level affects the accuracy of prediction of growth rate since the hazard of dying at age x determines the number of deaths in age groups (S2 Text). In the proposed algorithm, the life expectancy of reference populations can be set between 18 and 80 years according to the assumed mortality level in the population represented by the skeletal sample under study. To achieve such a flexibility, we adopted the reference age patterns of mortality from the Coale and Demeny West model life tables [37]. The tables represent the average mortality schedules that are based on a high number of 130 mortality experiences recorded in 22 modern populations known to have relatively good vital statistics. Other model life table systems that have been used in palaeodemographic studies involve Ledermann’s life tables [56] and logit systems by Brass [57] or by Ewbank et al. [58] based on either African [57] or pre-industrial standard life table [41]. Each of the model life table systems has its own strengths and weakness [15, 41], and there is no consensus as to which is the most appropriate for palaeodemography. Some authors experienced, however, that using the Coale and Demeny [37] and Brass [57] models, or other set of model tables makes little or no difference to results [47].

It has been suggested that model life tables are limited in the range of mortality patterns they can fit because they smooth out natural variation in the age-at-death distribution [1, 28, 54, 59]. Other approaches to the selection of reference demographic regimes involve using fixed set of life tables of historical or modern populations [12] or modern raw age-at-death data from the United Nation Database [24, 25].

All three reference mortality options (model life tables, life tables of modern populations, and modern raw age-at-death data) rely on the principle of demographic uniformitarianism [36]. The uniformitarian principle assumes that biological processes related to mortality and fertility respond similarly to variation in natural and cultural environment across human populations, and therefore modern demographic data can be interpolated to study the demography of prehistoric populations [30, 35, 36]. Uniformitarianism, however, does not imply that demographic rates do not change over time [36] but that demographic processes are constrained by the same biological processes across human populations [35]. We believe that the use of model life tables as a reference benefits from the variability of the mortality levels they span. The Coale and Demeny model life table covers almost the complete range of mortality levels that can be experienced by human populations (life expectancy at birth between 18 and 80 years). Both the modern reference life tables and modern age-at-death data from the United Nation Database encompasses a fixed and narrower range of life expectancy (18–38 years [12] and 42–74 years [24, 25]) and occupy a range of values of demographic parameters that may be outside the range assumed for the majority of prehistoric populations.

**Fig 6:**
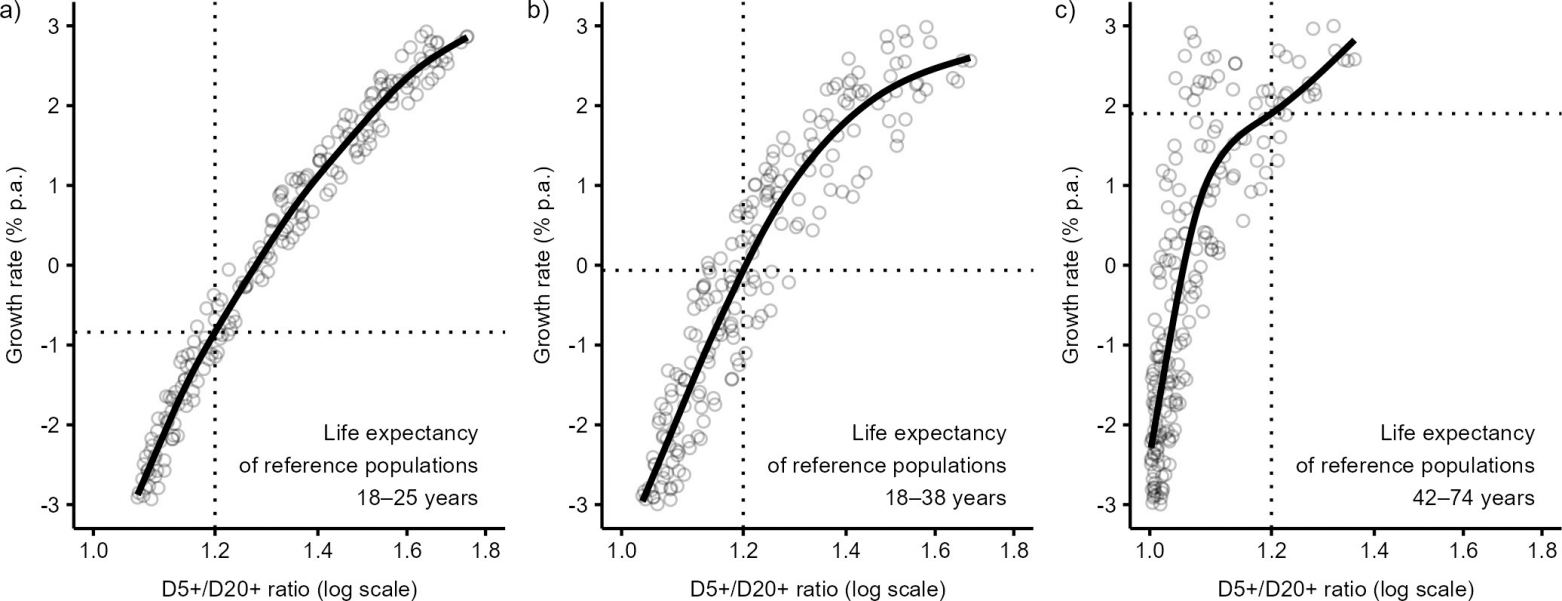
Relationship between the growth rate and the D5+/D20+ ratio (solid lines) in the sets of large-sized reference populations (circles) with the life expectancies at birth of (a) 18–25, (b) 18–38, and (c) 42–74 years. Mortality patterns of reference populations are based on the Coale and Demeny West model life tables with annual growth rate between −3 and 3%. The dotted lines depict the predicted growth rates in a population with the D5+/D20+ ratio of 1.2.

Fig 6 empirically documents how the choice of reference mortality level affects the prediction of the growth rate. The prediction lines describe the relationship between the growth rate and the D5+/D20+ ratio in three different sets of reference populations with life expectancies between (a) 18 and 25 (as used in this study), (b) 18 and 38 years [as used in 12], and (c) 42 and 74 years [as used in 24, 25]. Consider, for example, a skeletal sample with a D5+/D20+ ratio of 1.2, known to be derived from a population with the life expectancy between 18 and 25 years. The prediction based on the set of reference life tables with the same level of life expectancy (18–25 years, Fig 6A) led to the growth rate estimation of −0.8% per annum. The same methodology but based on the sets of reference populations with life expectancies between 18 and 38 years [as used in 12] gives the growth rate of −0.1% (Fig 6B) and prediction based on reference populations with life expectancies between 42 and 74 years [as used in 24] is 1.9% per annum (Fig 6C). The relationship between the reference mortality level and growth rate is inverse; if the fertility is constant, lower mortality is associated with higher growth. Therefore, the age-at-death ratio methods based on reference populations with lower mortality (higher life expectancy) than was that expected in population under study overestimate the true growth rate level and vice versa.

### Effect of stochastic variation on prediction of growth rate

**Fig 7:**
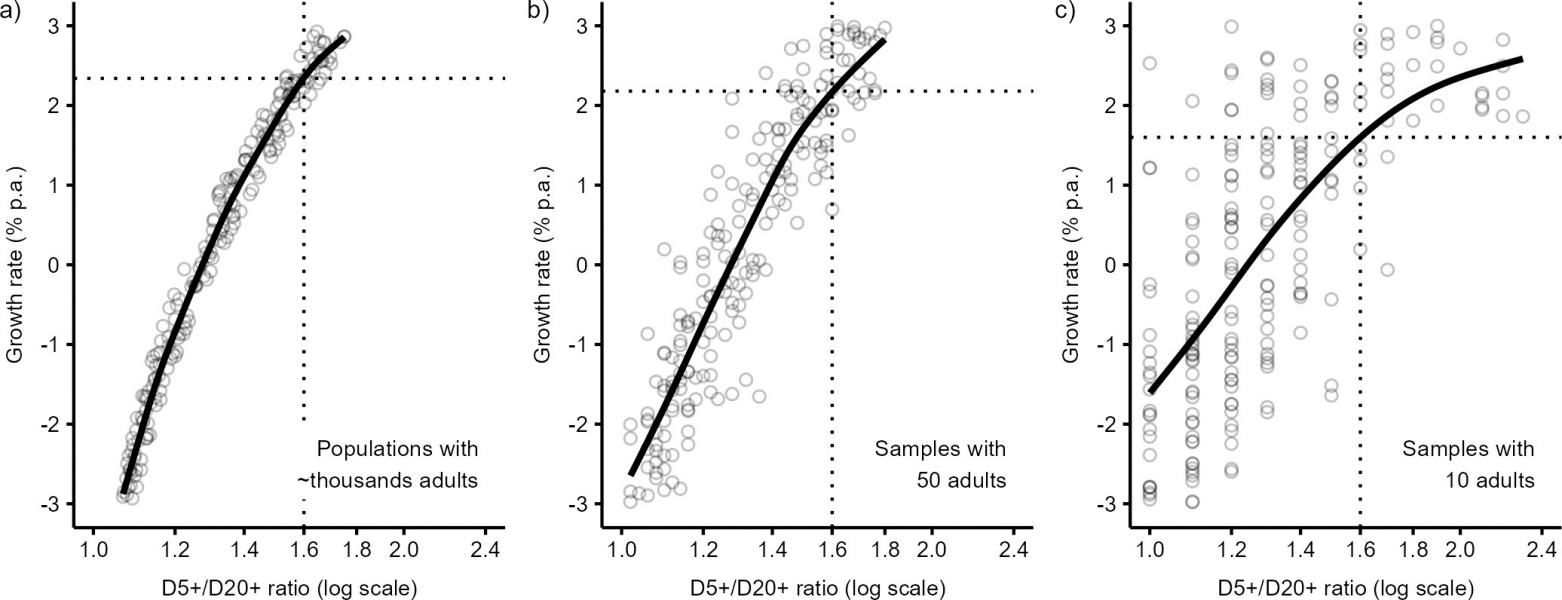
Relationship between the growth rate and the D5+/D20+ ratio (solid lines) in (a) large-sized reference populations (circles) and small-sized reference skeletal samples (circles) with (b) 50, and (c) 10 adult individuals. Mortality patterns of reference populations are based on the Coale and Demeny West model life tables with a life expectancy at birth between 18 and 25 years and annual growth rate between −3 and 3%. The dotted lines depict the estimated growth rates in the population or sample with the D5+/D20+ ratio of 1.6.

The second distinguishing feature of our ratio approach is that it reflects the effect of stochastic error due to sample size, which may be large in small skeletal samples and may affect the prediction. All previous studies on this subject allows predicting demographic rates from skeletal samples using prediction formulae derived from considerably larger reference samples of deaths. Fig 7 documents the effect of stochastic variation on demographic prediction by showing the relationship between growth rate and the D5+/D20+ ratio in the reference samples of various sizes. Fig 7A depicts the scatter and regression fit based on populations (or very large samples) with thousands of adult deaths, which is a good approximation for approaches calculating reference age-at-death data from age-specific mortality rates from life tables [e.g., 12] or adopting them directly from observed deaths in modern countries [24]. Fig 7B and 7C then show similar regression fit, but based on small skeletal samples with 50 and 10 adult deaths, which approximates settings of real skeletal samples. As expected, the distribution of the D5+/D20+ ratio has substantially higher variation in small samples than in large populations. Note that the maximum value of the D5+/D20+ ratio in large-sized populations is about 1.7, while in small samples there are ratios as large as 2.3.

Different distributions of the age-at-death ratio lead to different regression fits, and to different growth rate predictions. Fig 7 demonstrates, for example, that the D5+/D20+ ratio of 1.6 corresponds in a population based fit (Fig 7A) to a growth rate of ca 2.4%. By contrast, models based on small samples link the same ratio value with the growth rate of 2.2% and 1.6%, respectively. Fig 7 again demonstrates that the prediction of the growth rate may be inaccurate if it is performed on a reference set of samples with a number of adult skeletons that substantially differ from a number of adult skeletons of the sample under study. The distinctive feature of the presented algorithm is that skeletal samples are strictly compared to the reference set of simulated skeletal samples of the same size, which increases the accuracy of growth rate prediction.

The effect of stochastic variation on the age-at-death composition of skeletal samples is further related to the temporal scale of cemetery samples, which represent deaths accumulated over a longer period of time [1]. If we assume that population demographic rates did not change during burial activities at the site, then stochastic variation has the same effect regardless of the length of cemetery use. It does not depend on whether, for example, 100 individuals are buried in a single year or whether one individual is buried in each of the 100 years, because the demographic characteristics were constant. However, demographic rates tend to fluctuate over time and the skeletal samples therefore reflect the average demographic pattern over a longer period. The different temporal scale makes comparison of demographic estimates derived from different sources (ethnographic, historical, archaeological, and skeletal) less straightforward [4, 10, 30, 35]. Although long-term accumulation of skeletal samples may smooth out fluctuations in demographic behavior, the stochastic variation due to sample size still affects the age distribution of deaths and the prediction of average demographic patterns over a longer period of burial activities.

Given that the age-at-death composition of skeletal sample may be biased by stochastic variation and various natural and cultural factors, the ratio methods should be used to describe smoothed general trends in demographic rates over large areas and/or long time periods based on many samples [1, 21] rather than to predict demographic characteristics at the level of individual sites.

### Comparison with growth rate predictions based on Bocquet-Appel’s P index

There are six differences between our approach and Bocquet-Appel’s [12] approach. We used different (1) age-at-death ratio (D5+/D20+ vs. P index), (2) type of reference demographic regimes (Coale and Demeny model life tables vs. life tables of preindustrial populations), (3) range of life expectancy at birth (18–25 years vs. 18–38 years), (4) range of population growth (−3–3% vs. −2.5–2.5%), (5) type of regression (generalized additive model vs. power regression model), and (6) approach to accounting for stochastic error (yes vs. no).

**Fig 8:**
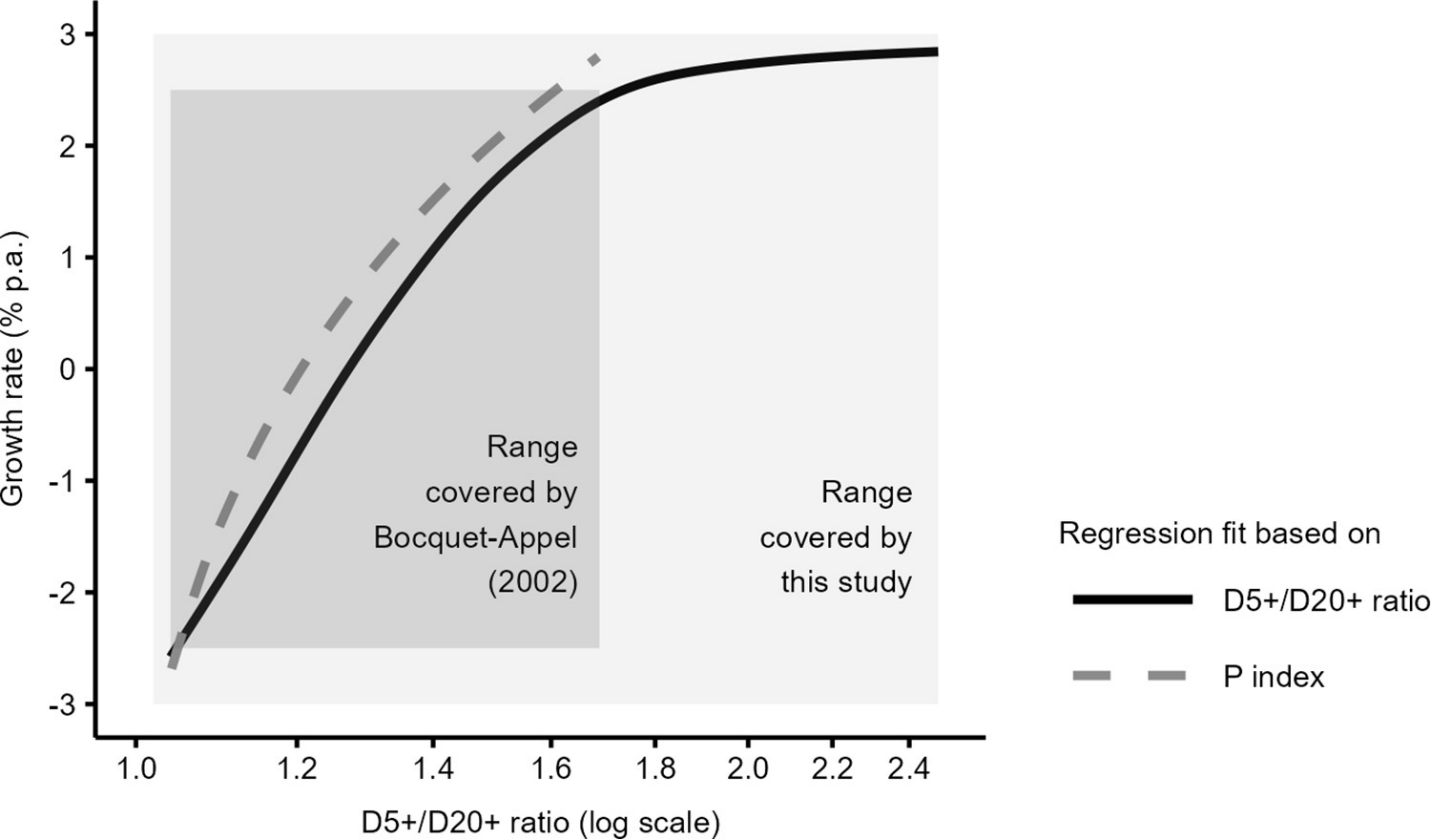
Comparison of regression fits of annual growth rate (%) as a function of the D5+/D20+ ratio (solid line) and P index (dashed line). The P index values have been converted to D5+/D20+ ratio values to allow the comparison in the same plot. Rectangles show the range of age-at-death ratio and growth rate observed in Bocquet-Appel’s [12] reference populations (dark gray) and in the set of reference skeletal samples used in our study (light gray).

Fig 8 demonstrates that the joint effect of these six factors has an effect on the position of the regression prediction lines, which explains the differences between the growth rate prediction based on our algorithm and Bocquet-Appel’s [12] method (as summarized in Fig 4). The systematic tendency of our approach to underestimate Bocquet-Appel’s [12] growth rate predictions can be mainly explained by the different levels of mortality assumed in the two methods (as documented in Fig 6) and by the existence of stochastic variation of the distribution of deaths (as documented in Fig 7), which Bocquet-Appel’s [12] method does not account for.

Fig 8 further shows that Bocquet-Appel reference populations cover a narrower window of the D5+/D20+ ratio distribution (ca. 1.1–1.7, see dark gray rectangle) than our approach does (ca. 1.0–2.5, see light gray rectangle). It means that Bocquet-Appel used his regression equation outside the range of the P index covered by reference populations since 10 out of his 68 skeletal samples have a D5+/D20+ ratio higher than 1.7 (equals to P index of 0.41). Prediction outside the range of reference data may, however, be invalid, as the relationship between the growth rate and the ratio can change outside this range. By contrast, our demographic predictions do not rest on extrapolation because the presented algorithm allows identifying cemetery samples with age-at-death ratios that are unlikely in skeletal samples of a given size and avoiding the estimation of demographic parameters based on such samples.

## Conclusions

Assuming that the age distribution of deaths reflects changes in fertility levels, we developed an algorithm for predicting growth, birth, and fertility rates in past populations from three age-at-death ratios (D5+/D20+, D3+/D20+, and D1+/D20+) quantifying the proportion of children and juveniles in skeletal samples. The methodology builds on the previous works [12, 22, 24] that proposed to apply a single prediction equation to skeletal samples from all time periods. The distinguishing feature of the proposed method is the algorithm by which the reference sets and prediction equations are constructed. The algorithm allows a customized regression model to be produced for each target skeletal sample. Each regression model is based on the unique reference set of simulated skeletal samples (1) that are derived from populations, whose life expectancies can be flexibly set to the level assumed in the time period represented by this target skeletal sample and (2) whose sizes match the size of the target skeletal sample. These new features reduce the effect of mortality bias and stochastic error and improve accuracy of the demographic prediction.

Our regression models for predicting growth and fertility rate from age-at-death ratio have a high explanatory power (≥83% when D20+≥50), despite the youngest children, the category most highly sensitive to changes in fertility, are excluded from the ratio computation. Due to stochastic variation, the prediction error is quite large when the estimate is based on small number of skeletons but decreases substantially with increasing sample size. The applicability of the proposed algorithm is illustrated by the comparison with the Bocquet-Appel’s [12] prediction equation, the most common method for predicting growth rate from distribution of deaths in skeletal samples.

## Supporting information

S1 Text R code for the simulation algorithm used in the prediction of demographic rates. (DOC)

S2 Text Effect of mortality pattern on the D5+/D20+ age-at-death ratio in terms of formal demography. (DOC)

S1 Table List of skeletal samples of the Bocquet-Appel dataset [12], including the chronological distance from the Neolithic front (dt), the total number of individuals (n), the numbers of individuals between 0 and 4 years (D0–4), between 5 and 19 years (D5–19), individuals older than 5 years (D5+) and older than 20 years (D20+), the D5+/D20+ age-at-death ratio and the P index, and the estimation of the growth rate (%), crude birth rate (CBR, the annual number of live births per 1,000 population), and total fertility rate (TFR, number of children per woman) based on the D5+/D20+ age-at-death ratio and the P index. (DOC)
