# Supplementary material for: A new method for estimating growth and fertility rates using age-at-death ratios in small skeletal samples: The effect of mortality and stochastic variation
Source: PLoS One. 2023 Jun 2;18(6):e0286580. doi: 10.1371/journal.pone.0286580 (PMC10237468; doi:10.1371/journal.pone.0286580)
Supplement: S1 Text — (DOC) [file pone.0286580.s001.doc]

S1 Text. R code for the simulation algorithm used in the prediction of demographic rates.

R code is available in R package demrat (https://github.com/galetap/demrat). Note that the package is available in the first released (0.1.2) at this stage (April, 6 2023).
