# Supplementary material for: A new method for estimating growth and fertility rates using age-at-death ratios in small skeletal samples: The effect of mortality and stochastic variation
Source: PLoS One. 2023 Jun 2;18(6):e0286580. doi: 10.1371/journal.pone.0286580 (PMC10237468; doi:10.1371/journal.pone.0286580)
Supplement: S2 Text — (DOC) [file pone.0286580.s002.doc]

S2 Text. Effect of mortality pattern on the D5+/D20+ age-at-death ratio in terms of formal demography.

Assuming stationary population and treating age (*x*) as a continuous variable, D5+ can be expressed as $\text{D5+}=\int_{5}^{\infty} l_{x}\cdot h(x)dx$, where $l_{x}$is the number of people at risk for dying at age *x*, and $h(x)$is the hazard of dying at age *x* . Similarly, D20+ can be expressed as $\text{D20+}=\int_{20}^{\infty} l_{x}\cdot h(x)dx$. Based on this formal description, it is clear, that the pattern of mortality $h(x)$affects the age-at-death ratio value. As $h(x)$ is independent of population growth, the same can be applied to stable populations.

Authors would like to thank anonymous reviewer of the earlier draft of this manuscript who drew our attention to the formal demographic aspect of the age-at-death ratios and proposed the above equations.

**References**

1. Milner GR, Boldsen JL. Population trends and the transition to agriculture: Global processes as seen from North America. Proc Natl Acad Sci. 2023; 120: e2209478119. https://doi.org/10.1073/pnas.2209478119
