# Supplementary material for: A new method for estimating growth and fertility rates using age-at-death ratios in small skeletal samples: The effect of mortality and stochastic variation
Source: PLoS One. 2023 Jun 2;18(6):e0286580. doi: 10.1371/journal.pone.0286580 (PMC10237468; doi:10.1371/journal.pone.0286580)
Supplement: S1 Table — (DOC) [file pone.0286580.s003.doc]

S1 Table. List of skeletal samples of the Bocquet-Appel dataset (2002), including the chronological distance from the Neolithic front (dt), the total number of individuals (n), the numbers of individuals between 0 and 4 years (D0–4), between 5 and 19 years (D5–19), individuals older than 5 years (D5+) and older than 20 years (D20+), the D5+/D20+ age-at-death ratio and the index P, and the predictions of the growth rate (%), crude birth rate (CBR, the annual number of live births per 1,000 population), and total fertility rate (TFR, number of children per woman) based on the D5+/D20+ age-at-death ratio and the index P. Predictions using the D5+/D20+ ratio are based on the sets of 500 simulated reference skeletal samples randomly drawn from populations with a life expectancy between 18 and 25 years and annual growth rate between −3 and 3%.

|  |  |  | Number of individuals | | | | |  | Age-at-death ratio | |  | Prediction based on D5+/D20+ ratio | | |  | Prediction based on P index | |
| --- | --- | --- | --- | --- | --- | --- | --- | --- | --- | --- | --- | --- | --- | --- | --- | --- | --- |
| Site | dt |  | n | D0–4 | D5–19 | D5+ | D20+ |  | D5+/D20+ | P |  | Growth | CBR | TFR |  | Growth | CBR |
| Aisne series | 100 |  | 50 | 10.0 | 15.0 | 40.0 | 25.0 |  | 1.600 | 0.375 |  | 1.9 | 72.9 | 10.0 |  | 2.5 | 67.8 |
| Aiterhofen Odmuhle | 100 |  | 142 | 5.4 | 21.4 | 136.6 | 115.2 |  | 1.186 | 0.157 |  | −1.0 | 35.9 | 4.5 |  | −0.2 | 33.2 |
| Ajdovska Jama | 1106 |  | 25 | 6.0 | 8.0 | 19.0 | 11.0 |  | 1.727 | 0.421 |  | 1.9 | 74.0 | 10.2 |  | 2.9 | 74.7 |
| Aven de la Boucle | 2324 |  | 60 | 3.2 | 7.8 | 56.8 | 49.0 |  | 1.159 | 0.137 |  | −1.1 | 35.1 | 4.4 |  | −0.5 | 29.9 |
| Bade-Wutemberg^a^ | 150 |  | 31 | 7.2 | 12.8 | 23.8 | 11.0 |  | 2.164 | 0.538 |  |  |  |  |  | 3.9 | 92.0 |
| Baume Bourbon 2 | 800 |  | 15 | 1.8 | 3.9 | 13.2 | 9.3 |  | 1.419 | 0.295 |  | 0.8 | 57.8 | 7.3 |  | 1.6 | 55.5 |
| Belleville | 2352 |  | 141 | 9.8 | 35.7 | 131.2 | 95.6 |  | 1.373 | 0.272 |  | 0.9 | 58.9 | 7.6 |  | 1.3 | 51.8 |
| Breuil-en-Vexin | 2200 |  | 88 | 28.0 | 20.0 | 60.0 | 40.0 |  | 1.500 | 0.333 |  | 1.6 | 68.7 | 9.3 |  | 2.0 | 61.4 |
| Brochtorff Circle | 2000 |  | 72 | 2.0 | 15.0 | 70.0 | 55.0 |  | 1.273 | 0.214 |  | 0.1 | 49.0 | 6.1 |  | 0.6 | 42.6 |
| Bruchstedt | 150 |  | 61 | 8.2 | 18.8 | 52.8 | 34.0 |  | 1.553 | 0.356 |  | 1.8 | 72.4 | 9.9 |  | 2.3 | 64.9 |
| Cabeco da Arruda | 1000 |  | 71 | 7.0 | 11.0 | 64.0 | 53.0 |  | 1.208 | 0.172 |  | −0.7 | 40.2 | 5.0 |  | 0.0 | 35.7 |
| Cala Colombo | 2650 |  | 24 | 4.0 | 6.3 | 20.0 | 13.8 |  | 1.455 | 0.313 |  | 1.0 | 61.5 | 7.9 |  | 1.8 | 58.2 |
| Calle Sant Pau^a^ | 1750 |  | 26 | 9.0 | 10.0 | 17.0 | 7.0 |  | 2.429 | 0.588 |  |  |  |  |  | 4.4 | 99.3 |
| Casa da Moura | 1900 |  | 214 | 27.0 | 61.0 | 187.0 | 126.0 |  | 1.484 | 0.326 |  | 1.7 | 69.6 | 9.4 |  | 2.0 | 60.3 |
| Cauna de Belesta 7 | 886 |  | 32 | 6.0 | 11.0 | 26.0 | 15.0 |  | 1.733 | 0.423 |  | 2.0 | 74.2 | 10.3 |  | 2.9 | 75.0 |
| Central Portugal | 3100 |  | 130 | 11.0 | 16.0 | 119.0 | 103.0 |  | 1.155 | 0.134 |  | −1.4 | 32.0 | 4.0 |  | −0.6 | 29.4 |
| Cerro Ortega | 3400 |  | 19 | 2.0 | 5.0 | 17.0 | 12.0 |  | 1.417 | 0.294 |  | 0.9 | 59.5 | 7.5 |  | 1.6 | 55.3 |
| Chamblandes | 500 |  | 116 | 25.0 | 24.0 | 91.0 | 67.0 |  | 1.358 | 0.264 |  | 0.7 | 57.2 | 7.3 |  | 1.3 | 50.5 |
| Columnata | −2350 |  | 114 | 50.4 | 16.6 | 63.6 | 47.0 |  | 1.354 | 0.261 |  | 0.7 | 57.1 | 7.3 |  | 1.2 | 50.1 |
| Cova de Avellaner | 729 |  | 19 | 4.0 | 4.0 | 15.0 | 11.0 |  | 1.364 | 0.267 |  | 0.6 | 57.2 | 7.2 |  | 1.3 | 51.0 |
| Dedeleben | 2433 |  | 23 | 2.6 | 9.4 | 20.4 | 11.0 |  | 1.856 | 0.461 |  | 2.1 | 77.2 | 10.8 |  | 3.3 | 80.7 |
| Derenburg | 2433 |  | 60 | 10.8 | 17.2 | 49.2 | 32.0 |  | 1.538 | 0.350 |  | 1.6 | 70.2 | 9.5 |  | 2.2 | 63.9 |
| Diconche | 2908 |  | 27 | 1.0 | 2.0 | 26.0 | 24.0 |  | 1.083 | 0.077 |  | −1.6 | 29.8 | 3.8 |  | −1.7 | 19.4 |
| Djerdap | −630 |  | 187 | 53.0 | 22.0 | 134.0 | 112.0 |  | 1.196 | 0.164 |  | −0.9 | 37.5 | 4.7 |  | −0.1 | 34.4 |
| Eybral | 3200 |  | 75 | 11.0 | 14.0 | 64.0 | 50.0 |  | 1.280 | 0.219 |  | 0.1 | 48.4 | 6.1 |  | 0.7 | 43.4 |
| Fontenay del Marmio | 800 |  | 62 | 9.3 | 16.7 | 52.7 | 36.0 |  | 1.463 | 0.316 |  | 1.7 | 69.3 | 9.4 |  | 1.9 | 58.8 |
| Gours aux Lions 2 | 2352 |  | 54 | 11.0 | 11.0 | 43.0 | 32.0 |  | 1.344 | 0.256 |  | 0.6 | 56.2 | 7.2 |  | 1.2 | 49.3 |
| Grossbrembach | 3000 |  | 105 | 18.5 | 28.2 | 86.6 | 58.3 |  | 1.484 | 0.326 |  | 1.7 | 69.4 | 9.4 |  | 2.0 | 60.3 |
| Hazleton North | 358 |  | 40 | 3.0 | 16.0 | 37.0 | 21.0 |  | 1.762 | 0.432 |  | 2.3 | 80.2 | 11.5 |  | 3.0 | 76.4 |
| Heidelsheim^a^ | 2050 |  | 21 | 4.3 | 9.8 | 16.7 | 6.9 |  | 2.420 | 0.587 |  |  |  |  |  | 4.3 | 99.1 |
| Jungsteinzeit | 3000 |  | 94 | 10.0 | 20.0 | 84.0 | 64.0 |  | 1.313 | 0.238 |  | 0.4 | 52.6 | 6.7 |  | 0.9 | 46.5 |
| La Clape 8^a^ | 3000 |  | 29 | 7.0 | 14.0 | 22.0 | 8.0 |  | 2.750 | 0.636 |  |  |  |  |  | 4.7 | 106.3 |
| Laris Groguet | 1900 |  | 110 | 16.0 | 16.0 | 94.0 | 78.0 |  | 1.205 | 0.170 |  | −0.8 | 39.1 | 4.9 |  | 0.0 | 35.4 |
| Lenzburg | 500 |  | 76 | 11.0 | 25.9 | 65.0 | 39.1 |  | 1.661 | 0.398 |  | 2.0 | 75.8 | 10.6 |  | 2.7 | 71.3 |
| Les Mournouards 2 | 2874 |  | 60 | 9.2 | 19.8 | 50.8 | 31.0 |  | 1.638 | 0.389 |  | 2.1 | 77.7 | 10.9 |  | 2.6 | 69.9 |
| Loisy en Brie | 2596 |  | 164 | 19.0 | 31.0 | 145.0 | 114.0 |  | 1.272 | 0.214 |  | 0.0 | 47.7 | 6.0 |  | 0.6 | 42.6 |
| Maillets | 2416 |  | 42.5 | 5.0 | 4.5 | 37.5 | 33.0 |  | 1.136 | 0.120 |  | −1.4 | 32.0 | 4.0 |  | −0.8 | 26.9 |
| Malesherbes-Orville | 350 |  | 24 | 1.0 | 5.5 | 23.0 | 17.5 |  | 1.314 | 0.239 |  | 0.5 | 54.2 | 6.8 |  | 0.9 | 46.6 |
| Moita do Sebastiao | −1250 |  | 136 | 22.7 | 17.3 | 113.3 | 96.0 |  | 1.181 | 0.153 |  | −1.0 | 35.9 | 4.5 |  | −0.3 | 32.5 |
| Monte Canelas 1 | 1880 |  | 147 | 25.0 | 25.0 | 122.0 | 97.0 |  | 1.258 | 0.205 |  | −0.1 | 45.7 | 5.7 |  | 0.5 | 41.1 |
| Montigny-Esb | 2800 |  | 111 | 6.0 | 26.0 | 105.0 | 79.0 |  | 1.329 | 0.248 |  | 0.6 | 54.7 | 7.0 |  | 1.1 | 48.0 |
| Moragy B 1 | 1050 |  | 81 | 16.2 | 25.8 | 64.8 | 39.0 |  | 1.662 | 0.398 |  | 2.0 | 75.8 | 10.6 |  | 2.7 | 71.3 |
| Niederbosa | 2281 |  | 93 | 14.4 | 28.6 | 78.6 | 50.0 |  | 1.572 | 0.364 |  | 2.0 | 74.3 | 10.3 |  | 2.4 | 66.1 |
| Nitra | 300 |  | 72 | 12.0 | 13.0 | 60.0 | 47.0 |  | 1.277 | 0.217 |  | 0.1 | 48.2 | 6.0 |  | 0.7 | 43.0 |
| Nordhausen^a^ | 3000 |  | 50 | 3.0 | 23.0 | 47.0 | 24.0 |  | 1.958 | 0.489 |  |  |  |  |  | 3.5 | 84.9 |
| Octrois | 200 |  | 41 | 2.0 | 6.0 | 39.0 | 33.0 |  | 1.182 | 0.154 |  | −1.0 | 37.0 | 4.6 |  | −0.3 | 32.7 |
| Paradis | 1724 |  | 17 | 2.0 | 7.5 | 15.0 | 7.5 |  | 2.000 | 0.500 |  | 2.2 | 76.5 | 10.9 |  | 3.6 | 86.5 |
| Pech 1 | 2650 |  | 42 | 7.0 | 7.0 | 35.0 | 28.0 |  | 1.250 | 0.200 |  | −0.2 | 45.4 | 5.6 |  | 0.4 | 40.3 |
| Pierre Folle | 2650 |  | 40 | 5.0 | 11.0 | 35.0 | 24.0 |  | 1.458 | 0.314 |  | 1.4 | 66.2 | 8.8 |  | 1.8 | 58.4 |
| Pontcharaud 2 | 803 |  | 98 | 21.0 | 22.4 | 77.0 | 54.6 |  | 1.411 | 0.291 |  | 1.2 | 64.1 | 8.4 |  | 1.6 | 54.8 |
| Reaudins | 550 |  | 39 | 8.0 | 7.0 | 31.0 | 24.0 |  | 1.292 | 0.226 |  | 0.3 | 51.6 | 6.4 |  | 0.8 | 44.5 |
| Rutzing Haid | 150 |  | 11 | 0.0 | 2.0 | 11.0 | 9.0 |  | 1.222 | 0.182 |  | −0.3 | 44.5 | 5.4 |  | 0.2 | 37.3 |
| Sammelserie | 150 |  | 81 | 6.6 | 15.4 | 74.4 | 59.0 |  | 1.261 | 0.207 |  | −0.1 | 46.6 | 5.8 |  | 0.5 | 41.5 |
| Schonstedt | 2300 |  | 64 | 17.1 | 18.9 | 46.9 | 28.0 |  | 1.675 | 0.403 |  | 2.2 | 78.2 | 11.1 |  | 2.7 | 72.0 |
| Skateholm | −1100 |  | 58 | 6.0 | 8.0 | 52.0 | 44.0 |  | 1.182 | 0.154 |  | −0.9 | 37.2 | 4.6 |  | −0.3 | 32.7 |
| Sondershausen | 150 |  | 47 | 4.2 | 10.8 | 42.8 | 32.0 |  | 1.338 | 0.252 |  | 0.6 | 55.6 | 7.1 |  | 1.1 | 48.7 |
| Stuttgart | 250 |  | 82 | 3.6 | 20.4 | 78.4 | 58.0 |  | 1.352 | 0.260 |  | 0.7 | 56.9 | 7.3 |  | 1.2 | 50.0 |
| Taforalt | −3500 |  | 179 | 78.0 | 21.0 | 101.0 | 80.0 |  | 1.263 | 0.208 |  | −0.1 | 46.4 | 5.8 |  | 0.5 | 41.6 |
| Trebur | 456 |  | 105 | 6.2 | 17.4 | 98.8 | 81.4 |  | 1.214 | 0.176 |  | −0.7 | 39.8 | 5.0 |  | 0.1 | 36.4 |
| Vedbaek | −100 |  | 23 | 5.0 | 5.0 | 18.0 | 13.0 |  | 1.385 | 0.278 |  | 0.7 | 57.8 | 7.3 |  | 1.4 | 52.7 |
| Vedrovice | 0 |  | 104 | 15.0 | 12.0 | 89.0 | 77.0 |  | 1.156 | 0.135 |  | −1.3 | 33.0 | 4.1 |  | −0.6 | 29.5 |
| Vikletice | 2700 |  | 141 | 22.5 | 28.5 | 118.5 | 90.0 |  | 1.317 | 0.241 |  | 0.5 | 53.4 | 6.8 |  | 1.0 | 46.8 |
| Villaine | 2900 |  | 135 | 21.0 | 17.3 | 114.0 | 96.7 |  | 1.179 | 0.152 |  | −1.1 | 35.2 | 4.4 |  | −0.3 | 32.4 |
| Villanykovesd | 1050 |  | 24 | 5.0 | 5.0 | 19.0 | 14.0 |  | 1.357 | 0.263 |  | 0.6 | 55.9 | 7.0 |  | 1.2 | 50.4 |
| Vilnyanka | 985 |  | 48 | 3.0 | 18.0 | 45.0 | 27.0 |  | 1.667 | 0.400 |  | 2.1 | 74.8 | 10.4 |  | 2.7 | 71.5 |
| Wandersleben | 2588 |  | 216 | 38.0 | 60.0 | 178.0 | 118.0 |  | 1.508 | 0.337 |  | 1.8 | 71.2 | 9.7 |  | 2.1 | 62.0 |
| Yasinovatka | 145 |  | 64 | 0.0 | 16.0 | 64.0 | 48.0 |  | 1.333 | 0.250 |  | 0.7 | 55.6 | 7.1 |  | 1.1 | 48.4 |
| Zengovarkony | 1050 |  | 64 | 3.0 | 5.0 | 61.0 | 56.0 |  | 1.089 | 0.082 |  | −2.0 | 26.1 | 3.4 |  | −1.6 | 20.3 |
| ^a^D5+/D20+ ratio is out of limits of its distribution in simulated reference samples. Estimation might be biased. | | | | | | | | | | | | | | | | | |

**References**

1. Bocquet-Appel J-P. Paleoanthropological traces of a Neolithic demographic transition. Curr Anthropol. 2002; 43: 637–650. https://doi.org/10.1086/342429
